# Exosomes Derived from *Dermatophagoides farinae* Induce Allergic Airway Inflammation

**DOI:** 10.1128/spectrum.05054-22

**Published:** 2023-06-14

**Authors:** Ting Yang, Zuyu Xu, Jinyan Yu, Jiaxi Liu, Wei Wang, Shanchao Hong

**Affiliations:** a Department of Dermatology, Affiliated Children’s Hospital of Jiangnan University, Wuxi, Jiangsu, People’s Republic of China; b Graduate School of Nanjing Medical University, Nanjing, Jiangsu, People’s Republic of China; c National Health Commission Key Laboratory on Parasitic Disease Prevention and Control, Jiangsu Provincial Key Laboratory on Parasites and Vector Control Technology, Jiangsu Institute of Parasitic Diseases, Wuxi, Jiangsu, People’s Republic of China; d Department of Clinical Laboratory, Jiangnan University Medical Center, Wuxi, Jiangsu, People’s Republic of China; Hubei University of Medicine

**Keywords:** *Dermatophagoides farinae*, exosome, allergic airway inflammation, bronchial epithelial cell, alveolar macrophage, comparative transcriptomics

## Abstract

House dust mites (HDMs) are a major source of indoor allergens that cause airway allergic disease. *Dermatophagoides farinae*, a predominant species of HDMs in China, has demonstrated pathogenic role in allergic disorders. Exosomes derived from human bronchoalveolar lavage fluid have been strongly associated with allergic respiratory diseases progression. However, the pathogenic role of *D. farinae*-derived exosomes in allergic airway inflammation has remained unclear until now. Here, *D. farinae* was stirred overnight in phosphate-buffered saline, and the supernatant was used to extract exosomes by ultracentrifugation. Then, shotgun liquid chromatography-tandem mass spectrometry and small RNA sequencing were performed to identify proteins and microRNAs contained in *D. farinae* exosomes. Immunoblotting, Western blotting, and enzyme-linked immunosorbent assay demonstrated the specific immunoreactivity of *D. farinae*-specific serum IgE antibody against *D. farinae* exosomes, and *D. farinae* exosomes were found to induce allergic airway inflammation in a mouse model. In addition, *D. farinae* exosomes invaded 16-HBE bronchial epithelial cells and NR8383 alveolar macrophages to release the inflammation-related cytokines interleukin-33 (IL-33), thymic stromal lymphopoietin, tumor necrosis factor alpha, and IL-6, and comparative transcriptomic analysis of 16-HBE and NR8383 cells revealed that immune pathways and immune cytokines/chemokines were involved in the sensitization of *D. farinae* exosomes. Taken together, our data demonstrate that *D. farinae* exosomes are immunogenic and may induce allergic airway inflammation via bronchial epithelial cells and alveolar macrophages.

**IMPORTANCE** *Dermatophagoides farinae*, a predominant species of house dust mites in China, has displayed pathogenic role in allergic disorders, and exosomes derived from human bronchoalveolar lavage fluid have been strongly associated with allergic respiratory diseases progression. However, the pathogenic role of *D. farinae*-derived exosomes in allergic airway inflammation has remained unclear until now. This study, for the first time, extracted exosomes from *D. farinae*, and sequenced their protein cargo and microRNAs using shotgun liquid chromatography-tandem mass spectrometry and small RNA sequencing. *D. farinae*-derived exosomes trigger allergen-specific immune responses and present satisfactory immunogenicity, as revealed by immunoblotting, Western blotting, and enzyme-linked immunosorbent assay and may induce allergic airway inflammation via bronchial epithelial cells and alveolar macrophages. Our data provide insights into the mechanisms of allergic airway inflammation caused with *D. farinae*-derived exosomes and the treatment of house dust mite-induced allergic airway inflammation.

## INTRODUCTION

Human dust mites (HDMs) have been widely accepted as a major source of allergens associated with asthma and other allergic disorders (1). Currently, the major species of HDMs include Dermatophagoides pteronyssinus, Dermatophagoides farinae, Euroglyphus maynei, and Blomia tropicalis, with the former two being ubiquitous in temperate and tropical regions (2–4).

*D. farinae* is the predominant species in China and contains a large number of allergens, which can cause allergic diseases. The pathogenic role of *D. farinae* in allergic conditions has been extensively investigated (5). In NC/Nga mice, intranasal administration of crude extract of *D. farinae* resulted in allergic asthma-like responses, such as goblet cell hyperplasia, pulmonary eosinophilic inflammation, and an increase in total serum IgE and *D. farinae*-specific IgG1 antibodies (6). The *D. farinae* allergen Der f 31 was identified to activate epithelial cells and enhance lung-resident group 2 innate lymphoid cells (ILC2s) (7). Repeated exposure to *D. farinae* allergens caused neutrophil-dominant airway inflammation together with fibrotic changes and formation of lymphoid clusters in a dose- and frequency-dependent manner, and secretion of type 1 and type 2 inflammatory cytokines was also induced (8). Mite feces, with particles 20 to 30 μm in diameter, are primary airborne allergens associated with asthma (9, 10). Particles of ≤5 μm would be inhaled into bronchioles more easily. In addition, there is a progressive decrease in the percentages of particles entering the peripheral lung with increasing size (11).

Exosomes are small, single-membrane, secreted organelles 30 to 150 nm in diameter that enable cell-to-cell communication by shuttling different molecules, such as nucleic acids, lipids, proteins, and glycoconjugates (12). Previous studies have shown the potential of exosomes as promising noninvasive diagnostic and therapeutic targets (13–15). Through release of their cargo, exosomes derived from human bronchoalveolar lavage fluid (BALF) have been strongly associated with allergic respiratory disease progression (16). In addition, serum-derived exosomes from HDM-sensitized guinea pigs were found to play a proinflammatory role in bronchial epithelial BEAS-2B cells (17). However, the pathogenic role of *D. farinae*-derived exosomes in allergic airway inflammation has remained unclear until now.

In this study, we extracted and identified exosomes from *D. farinae* and sequenced its proteins and microRNAs (miRNAs) using shotgun liquid chromatography-tandem mass spectrometry (LC-MS/MS) and small RNA sequencing (sRNA-Seq). Then, we used a mouse model of allergic airway inflammation and serum samples from *D. farinae* allergic patients to demonstrate the sensitization and immunogenicity of *D. farinae* exosomes. Furthermore, we focused on human bronchial epithelial cells and rat alveolar macrophages to investigate the role of *D. farinae* exosomes in allergic airway inflammation.

## RESULTS

### Characterization of *D. farinae*.

**FIG 1:**
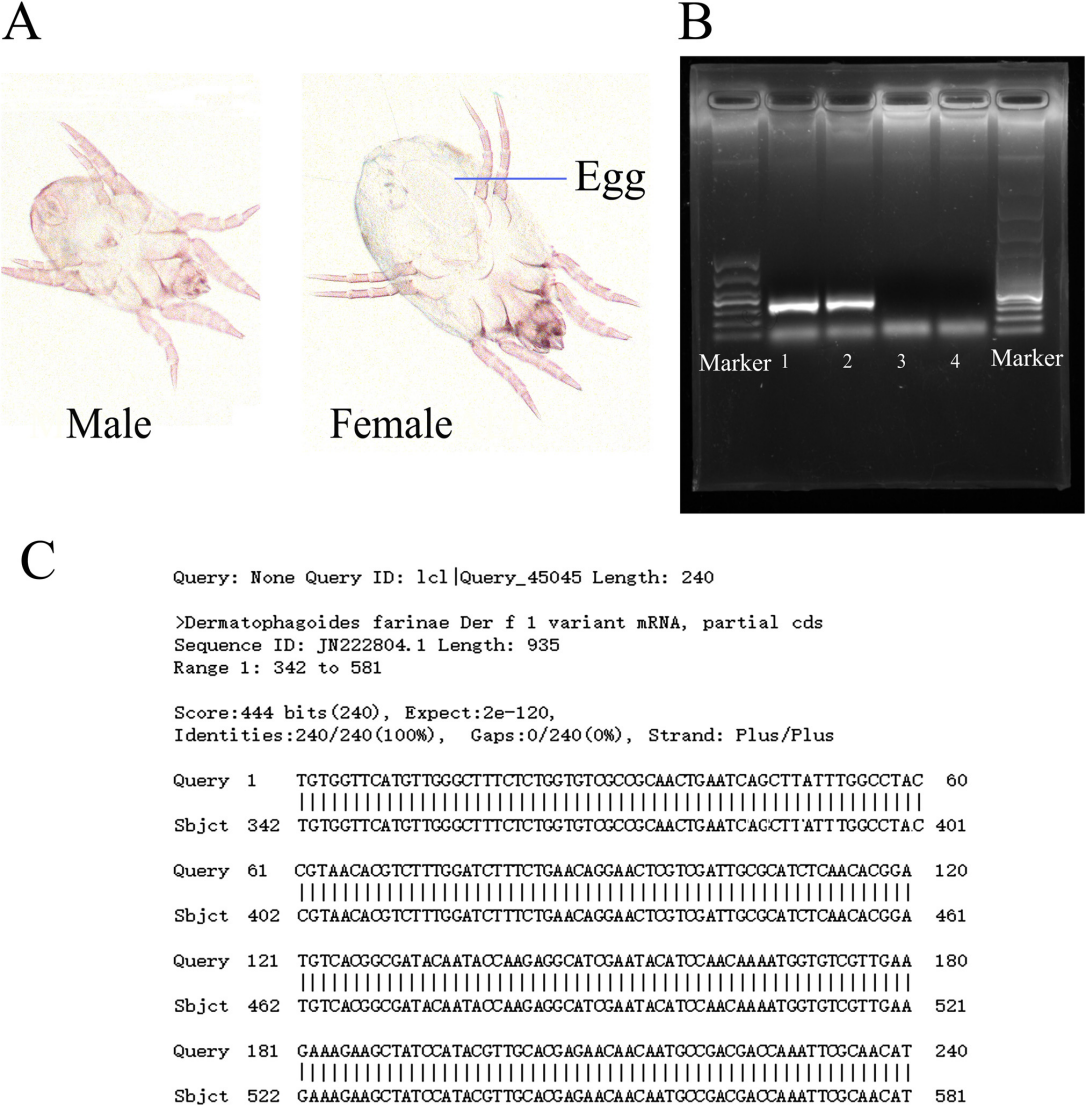
Identification of *Dermatophagoides farinae* purity. (A) Photomicrograph of adult male and female *D. farinae*. (B) Der f 1 was detected by nest PCR assay from *D. farinae* cultured in laboratory (lanes 1 and 2). Der p 1 was not detected from the same template (lanes 3 and 4). (C) Sequence of PCR amplification product from lanes 1 and 2 in panel B.

Morphologic characterization distinguished HDMs from storage mites (Fig. 1A), and a PCR assay confirmed no contamination from *D. pteronyssinus* (Fig. 1B). The amplification region was sequenced with a 100% homology with *D. farinae Der f 1 mRNA* (Fig. 1C). These data indicate that the subsequent extracted exosomes were all derived from *D. farinae*.

### Characterization of *D. farinae* exosomes.

**FIG 2:**
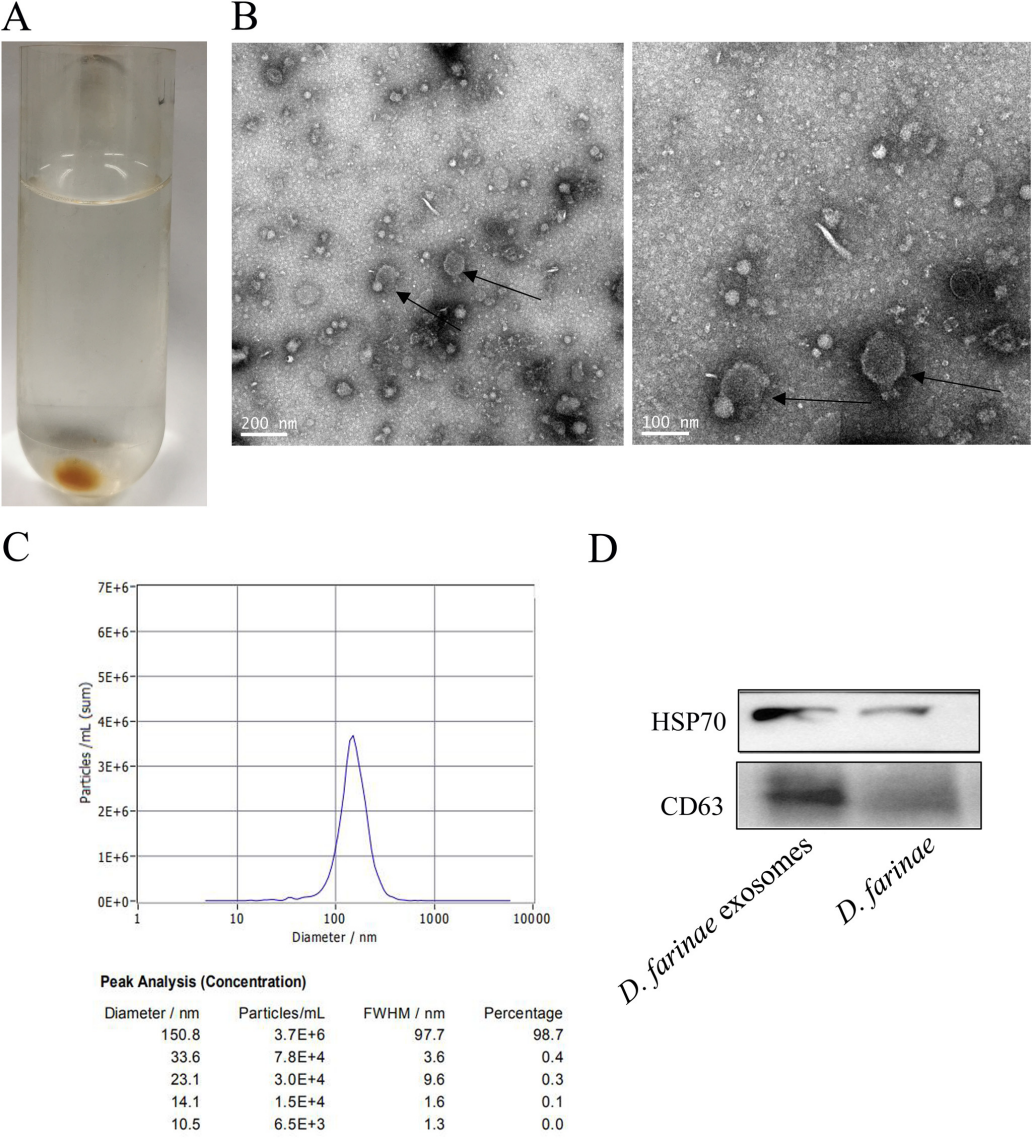
Characterization of *D. farinae* exosomes. (A) Light-yellow gel-like pellet is acquired after ultracentrifugation. Transmission electron microscopy (B), nanoparticle tracking analysis (C), and Western blotting (D) were used to detect typical characteristics of *D. farinae* exosomes.

After ultracentrifugation of the supernatant acquired by *D. farinae* that was stirred overnight, light-yellow gel-like precipitates were produced (Fig. 2A). Transmission electron microscopy showed that the isolated *D. farinae* extracellular vesicles had characteristic cup-shaped and lipid bilayer morphology (Fig. 2B). Nanoparticle tracking analysis of *D. farinae* exosomes showed a mean diameter of 150 nm with a 10^9^/mL particle concentration (Fig. 2C). In addition, Western blotting detected CD63 at 23 kDa and HSP70 at 70 kDa in *D. farinae* exosomes (Fig. 2D).

### Protein and miRNA expression profiles in *D. farinae* exosomes.

Shotgun LC-MS/MS was performed to identify the protein expression profile in *D. farinae* exosomes, and proteins with unique peptides of ≥1 were considered reliable for high-precision Q EXactive series mass spectrometry. *D. farinae* was used as keywords in UniProt to blast the peptide sequence, and a total of 72 proteins were retrieved (Table 1). Among these proteins, there were 55 *D. farinae* proteins, and the other 17 proteins belonged to a *Cardinium endosymbiont* of *D. farinae*. Among these 55 *D. farinae* proteins, 29 proteins have been recognized by the World Health Organization/International Union of Immunological Societies (WHO/IUIS) Allergen Nomenclature Subcommittee and have been associated with allergic symptoms, and 6 proteins (Allergen, MAG29, Alpha-actinin, Zen 1 protein, elongation factor 1, and Der f 9) have been associated with allergy (18–21). Astigmatid mites are reported to host intracellular bacteria, such as *Cardinium* and *Wolbachia* (22), and we detected *Cardinium* in *D. farinae* using PCR assay based on alignment of the *Cardinium 16S rRNA* gene sequences, but not in *D. pteronyssinus*, as reported previously (see Fig. S3 in the supplemental material) (23, 24). It has been demonstrated that the mite microbiome affects allergies by altering allergen production or directly producing substances, such as endotoxins or exoproteases (25). It is therefore hypothesized that these 17 proteins from *Cardinium* in *D. farinae* exosomes may also contribute to the allergenicity of *D. farinae*.

**TABLE 1:** Protein expression profile of *D. farinae* exosomes^*a*^

Organism and accession no.	Protein description	Coverage (%)	No. of peptides	No. of PSMs	No. of unique peptides
*Dermatophagoides farinae* 1^*b*^					
A1YW11	Der f 1 allergen	20	5	9	5
A7XXV2	Der f 2 allergen	51	7	18	1
A3F5F1	Der f 2 allergen	61	7	15	1
A1YW14	Der f 2 allergen	61	7	16	1
Q5TIW0	Der f 2 allergen	49	6	13	1
A1KXH3	Der f 3 allergen	18	4	5	4
A0A023NMA7	Der f 4 alpha-amylase	13	4	7	4
A1KXG5	Der f 5 allergen	24	5	7	5
B7U5T0	Der f 6 allergen	12	3	4	3
A1KXH4	Der f 7 allergen	24	7	9	4
A0A088SAS4	Der f 7 allergen	15	4	4	1
A1KXG7	Der f 7 allergen	39	5	8	5
L7V2G7	Der f 8 glutathione transferase	38	6	10	6
A0A088SAH7	Der f 8 glutathione transferase	41	7	7	7
A0A088SCQ8	Der f 9 allergen	35	7	9	7
Q23939	Der f 10 tropomyosin	47	15	21	15
A0A088SCQ4	Der f 11 allergen	19	13	14	13
Q9U6R7	Der f 11 98-kDa HDM allergen	21	9	11	9
Q1M2P5	Der f 13 allergen	60	7	11	7
A1KXH1	Der f 13 allergen	24	3	3	3
A0A2Z6DTI4	Der f 14 apolipophorin	60	99	264	99
Q8MVU3	Der f 16 allergen	11	5	5	5
Q86R84	Der f 18 60-kDa allergen	2	1	1	1
A0A088SAW4	Der f 20 arginine kinase	58	16	32	1
A7XZJ2	Der f 20 arginine kinase	48	15	26	11
B2GM84	Der f 21 mite allergen	52	7	12	7
A0A482J3M1	Der f 23 allergen	24	17	18	17
A0A7D4W6V2	Der f 24 cytochrome *c*-like protein	53	5	8	5
A0A088SAX2	Der f 25 triosephosphate isomerase	62	12	16	12
A0A088SV31	Der f 27 allergen	23	7	8	7
A0A088SAS1	Der f 28 allergen	6	3	5	1
A0A7D4W6U8	Der f 28 heat shock 70-like protein	26	4	4	4
I1ZE45	Der f 28 heat shock protein cognate 5	11	4	4	4
L7V065	Der f 28 heat shock protein 70	10	5	7	3
A1KXG2	Der f 29 peptidyl-prolyl *cis-trans* isomerase	26	4	6	4
A0A7D4W7J5	Der f 29	15	3	3	3
L7UZ91	Der f 30 ferritin	58	9	80	9
A0A088SAG5	Der f 30 allergen	48	7	11	7
A0A088SAY1	Der f 31 allergen	64	9	13	9
A0A088SV41	Der f 33 tubulin alpha chain	4	2	2	2
A0A1W7HBY9	Der f 35 group II allergen	22	2	2	2
A0A291KZC2	Der f 36 allergen	40	5	7	5
A0A411P9C6	Der f 38 bacterial lytic enzyme	18	1	1	1
A0A411P9C3	Der f 39 allergen	10	2	2	2
A5X5X4	Allergen	45	4	5	4
P39674	Allergen MAG29	43	6	11	6
L7UZ85	Alpha-actinin	1	1	1	1
A0A088SV45	Profilin	28	3	4	3
I7HDR2	Zen 1 protein	7	3	3	3
I1ZCC6	Elongation factor 1α	38	13	17	13
A0A5B8ZYM5	GAPDH	62	1	1	1
A0A5B9A1C0	Calreticulin	44	2	2	2
X4ZE83	2-Phospho-d-glycerate hydro-lyase	47	16	30	16
A1KXC3	Arginine kinase	56	16	27	1
A0A7D4W741	Aldehyde dehydrogenase-like protein	42	16	22	16
A0A482GMV4	Fructose-bisphosphate aldolase	35	13	19	13
A0A088SCP3	Inorganic diphosphatase	26	6	7	6
A0A7D4WQE4	Lysosomal aspartic protease-like protein	16	6	6	6
A0A088SAS8	Beta-*N*-acetylhexosaminidase	10	4	4	4
A1KXC1	DFP1	5	1	1	1
A0A5B9A2T8	60S ribosomal protein L11	39	1	1	1
A0A411P9B7	Petrotrophic-like protein	10	2	2	2
A0A1J1DL12	MAG133	16	2	2	2
A0A2C9PGE6	AQP1 aquaporin	8	2	2	2
A0A2I6AXE4	Argonaute 8	3	1	1	1
A0A2I6AXD5	Argonaute 7	1	1	1	1
A0A2I6AXE0	Argonaute 1	1	1	1	1
A0A2I6AXD7	Argonaute 4	1	1	1	1
*Dermatophagoides farinae* 2^*c*^					
A0A556QWM9	Uncharacterized protein GN = FPG78_03420	47	14	23	14
A0A556QWR3	Uncharacterized protein GN = FPG78_03425	71	25	44	25
A0A556QW25	PorT family protein GN = FPG78_01975	30	8	13	8
A0A556QXD2	Uncharacterized protein GN = FPG78_04955	31	11	13	11
A0A556QX46	OmpH family outer membrane protein GN = FPG78_04445 GN=FPG78_04445	31	11	13	11
A0A556QX47	ATP synthase subunit beta GN = atpD	5	2	2	2
A0A556QVK8	Probable cytosol aminopeptidase GN = pepA	9	3	3	3
A0A556QU82	Uncharacterized protein GN = FPG78_06180	4	1	1	1
A0A556QV83	Ankyrin repeat domain-containing protein GN=FPG78_00455	3	1	1	1
A0A556QVA2	Acyl carrier protein GN = acpP	15	1	1	1
A0A556QWL5	Methionine–tRNA ligase GN = metG	2	1	1	1
A0A556QU24	Virulence protein GN = FPG78_06540	1	1	1	1
A0A556QWN1	Alanine–tRNA ligase GN = FPG78_03435	2	1	1	1
A0A556QV62	Ankyrin repeat domain-containing protein GN=FPG78_00315	11	1	1	1
A0A556QWC7	tRNA [*N*(6)-l-threonylcarbamoyladenosine(37)-C(2)]-methylthiotransferase MtaB GN=mtaB	2	1	1	1
A0A556QXH3	Protein GrpE GN = grpE	5	1	1	1
A0A556QX70	AAA-ATPase_like domain-containing protein GN=FPG78_04535	5	1	2	1

a The accession number indicates the protein number in the FASTA database (https://www.uniprot.org/). Coverage indicates the percentage of the protein sequence covered by identified peptides. The peptide column indicates the number of distinct peptide sequences in the protein group. PSMs, peptide spectrum matches (i.e., the total number of identified peptide sequences for the protein, including those redundantly identified). Unique peptides refer to peptide sequences unique to a protein group.

b OX = 6,954.

c OX = 6,954; OS, cardinium endosymbiont of *Dermatophagoides farinae*.

sRNA-Seq was performed to investigate miRNAs expression profiles in *D. farinae* exosomes. We aligned sRNA to the miRNA of corresponding species in miRBase and identified 42 conserved miRNAs (Table 2). A total of 32 novel miRNAs were acquired by miRDeep2 according to mature sequences and predicted hairpin sequences of this species (Table 3). dfa-miR-276-3p, dfa-miR-7, dfa-miR-279, dfa-miR-1-3p, dfa-miR-5735-3p, dfa-miR-7-5p, and dfa-let-7b showed high expression levels (>1,000 transcripts per million), and these miRNAs have been reported to be highly expressed in other insect species. Among these miRNAs, dfa-miR-1-3p, dfa-miR-7, and dfa-let-7b, which were also detected in humans, have been found to be associated with a wide range of human diseases, including asthma (26–28). High expression of dfa-novel-miR1 to novel-miR9 was detected (>1,000 transcripts per million), and the expression of these novel miRNAs found in *D. farinae* exosomes were not lower relative to conserved miRNAs, which was not consistent with miRNAs sequencing results of *D. pteronyssinus* (29). This is probably because the materials we used for sequencing were *D. farinae* exosomes rather than HDMs.

**TABLE 2:** Conserved miRNA expression profile of *D. farinae* exosomes^*a*^

miRNA ID	UMI_TPM	Sequence of mature miRNA (5′–3′)	End Pos
dfa-miR-276-3p	2889.213986	UAGGAACUUCAUACCAUGCUCG	300
dfa-miR-7	2834.299534	UGGAAGACUAGUGAUUUUGUUG	51
dfa-miR-279	1503.293265	UGACUAGAUCCACACUCAUCC	300
dfa-let-7b	1396.425037	AACUAUACAACCUACUACCUCA	51
dfa-miR-1-3p	1311.457698	UGGAAUGUAAAGAAGUAUGGAG	271
dfa-miR-5735-3p	1105.143209	UGGACAACAGGAUAAUGGCGU	270
dfa-miR-7-5p	1093.665521	UGGAAGACUAGUGAUUUUGUUGU	51
dfa-miR-133-3p	974.5895743	UUGGUCCCCUUCAACCAGCUGU	271
dfa-miR-71c-5p	660.4740415	UGAAAGACAUGGGUAGUGAGAU	51
dfa-miR-279	628.1499512	UGACUAGAUCCACACUCAUCCA	300
dfa-miR-252	627.4199215	CUAAGUAGUAGUGCCGCAGGU	51
dfa-miR-375-3p	391.9853614	UUUGUUCGUUCGGCUCGAGUUA	51
dfa-miR-124-3p	334.59692	UAAGGCACGCGGUGAAUGCCA	270
dfa-miR-9-3p	258.9171803	AUAAAGCUAGGUUACCAAAGUUA	272
dfa-miR-133-3p	245.0060599	UUGGUCCCCUUCAACCAGCUG	270
dfa-miR-71	244.3977018	UGAAAGACAUGGGUAGUGA	51
dfa-miR-307a	234.9884309	UCACAACCUCCUUGAGUGAG	51
dfa-miR-252-5p	232.3522127	CUAAGUACUAGCGCCGCAGGAG	22
dfa-miR-71_2	219.9417088	UGAAAGACAUGGGUAGUGAGAUG	51
dfa-miR-276	179.0600487	UAGGAACUUCAUACCAUGCUC	300
dfa-miR-279a_1	112.9112516	UGACUAGAUCCACACUCAU	300
dfa-miR-124a	106.4626564	UAAGGCACGCGGUGAAUGCC	269
dfa-miR-9-5p_2	93.44379439	UCUUUGGUUAUCUAGCUGUAU	21
dfa-miR-993a-3p	58.64571471	GAAGCUCGUUUCUACAGGUUUC	300
dfa-miR-278-3p	53.90052202	UCGGUGGGAUUUUCGUCCGUC	270
dfa-miR-7-5p_6	40.67887403	UGGAAGACUAGUGAUUUUGUUGUU	51
dfa-miR-124-3p	38.4482279	UAAGGCACGCGGUGAAUGC	268
dfa-miR-137-3p_1	24.25320705	UUAUUGCUUGAGAAUACACG	269
dfa-miR-124-3p_3	13.66777722	UAAGGCACGCGGUGAAUGCCAA	271
dfa-miR-184-3p_3	9.895957392	UGGACGGAGAACUGAUAAGGGC	271
dfa-miR-9a-5p	8.598126914	UCUUUGGUUAUCUAGCUGUAUGA	23
dfa-miR-9-5p_1	8.395340902	CUUUGGUUAUCUAGCUGUAUGA	51
dfa-miR-184_1	8.151997688	UGGACGGAGAACUGAUAAGGG	270
dfa-miR-9-5p_5	6.570266793	UCUUUGGUUAUCUAGCUGUAUG	22
dfa-miR-124-3p_1	4.704635481	UAAGGCACGCGGUGAAUGCCAAG	272
dfa-miR-210_4	2.636218158	UUGUGCGUGUGACAGCGGCU	300
dfa-miR-7-5p_1	0.567800834	UGGAAGACUAGUGAUUUUGUUGUUC	51
dfa-miR-210a_1	0.486686429	UUGUGCGUGUGACAGCGGCUA	300
dfa-miR-210-3p_2	0.283900417	CUUGUGCGUGUGACAGCGGCUAU	300
dfa-miR-307-3p_1	0.202786012	UCACAACCUCCUUGAGUGAGUGA	51
dfa-miR-234	0.121671607	UUAUUGCUUGAGAAUACA	267
dfa-miR-307b-5p	0.040557202	UCACUCAAGGAGGUUGUGAUG	21

a UMI_TPM, unique molecular identifiers_transcript per million. End Pos, end position of mature miRNA on hairpin.

**TABLE 3:** Novel miRNA expression profile of *D. farinae* exosomes

Novel miRNA ID (dfa-)	Chromosome/strand	Position		Sequence (mature, 5′–3′)	UMI_TPM^*a*^
		Start	End		
Novel-miR1	NBAF01001692.1–	118166	118246	UAUCACAGCCUUUUUGAUGUCU	6535.712057
Novel-miR2	NBAF01001692.1–	118011	118069	UAUCACAGCCUAGUUAACACGAU	5605.857076
Novel-miR3	NBAF01001684.1+	336421	336486	UGAUAUGUUUGAUAUUCUUGGUU	4321.369918
Novel-miR4	NBAF01001692.1–	117667	117745	UAUCACAGCCACUUUGAUUAGU	4047.446573
Novel-miR5	NBAF01001646.1+	298262	298318	GCGCGAUUGGACCCGUGCUGACGUC	2702.366955
Novel-miR6	NBAF01001475.1+	27195	27279	AACACAUCUAGCUUGUAAGGAUU	2441.219128
Novel-miR7	NBAF01001483.1+	51249	51297	GAGAAAGGUGCCCGUCAAGUCU	1641.512211
Novel-miR8	NBAF01001680.1–	274699	274773	UAAGGCCUUUAUGUUUCGUAUGA	1465.777853
Novel-miR9	NBAF01001692.1–	117854	117916	UAUCACAGCCAGCUUUGGUGAGU	1077.604868
Novel-miR10	NBAF01001250.1–	1374	1450	AUAAGAUUUUGAAACGACAAGA	351.9148454
	NBAF01001703.1+	910296	910372	AUAAGAUUUUGAAACGACAAGA	
Novel-miR11	NBAF01001475.1+	24072	24153	UACGGUCCUCUUGUGUGCCUUU	312.3310159
Novel-miR12	NBAF01001648.1+	299020	299084	UUCGUAAUAAGUUUAACGGAC	114.006296
	NBAF01001114.1+	6136	6197	UUCGUAAUAAGUUUAACGGAC	
Novel-miR13	NBAF01001622.1+	220433	220495	UUGACUAGAACUCACCUUCGUA	110.0316902
Novel-miR14	NBAF01001513.1–	62072	62143	UUGUAUACUAAAGUGAGGAUCU	45.95131035
Novel-miR15	NBAF01001608.1+	146489	146564	UAAGAUAACUUAUUACGGUUGG	35.20365171
Novel-miR16	NBAF01001683.1–	178183	178233	UGCGAUCAUUUUGCAUUGUUGGUU	22.10367532
Novel-miR17	NBAF01001655.1+	327534	327603	UUCCAACAUUUGAACAUUUUAAG	9.855400189
Novel-miR18	NBAF01001113.1+	11997	12072	UGAUUGGCUCGUGGAUGUUACAUC	5.394107923
	NBAF01000726.1+	17084	17159	UGAUUGGCUCGUGGAUGUUACAUC	
	NBAF01001395.1+	28146	28221	UGAUUGGCUCGUGGAUGUUACAUC	
Novel-miR19	NBAF01001402.1+	12496	12544	UGGAUUCAGAGAUGUCGUACCAGU	10.42320102
Novel-miR20	NBAF01000990.1+	11868	11945	UGAUUUUAUUGUUUGAAUGUCGGU	2.879561372
	NBAF01001321.1+	21862	21939	UGAUUUUAUUGUUUGAAUGUCGGU	
	NBAF01000720.1+	7252	7329	UGAUUUUAUUGUUUGAAUGUCGGU	
Novel-miR21	NBAF01001601.1–	170460	170542	UCGAUGAAACUAGACAAUGAUGUU	1.460059287
Novel-miR22	NBAF01001656.1+	322036	322077	UGUAACUCGUUAGCGCUGU	2.798446967
	NBAF01001483.1+	47252	47293	UGUAACUCGUUAGCGCUGU	
	NBAF01001663.1+	21152	21193	UGUAACUCGUUAGCGCUGU	
Novel-miR23	NBAF01001517.1+	14268	14325	UUACGUAUUUUUUCCCGUUCGUUU	1.17615887
Novel-miR24	NBAF01001623.1+	215112	215156	GGAACCACGCUCUGCUACCA	0.811144049
Novel-miR25	NBAF01001403.1+	10223	10269	UGGUUACCAUUCGGUCGAGGUU	0.567800834
Novel-miR26	NBAF01001654.1–	76933	77000	UUGUAGUCGCACCGCCACCACC	0.608358036
	NBAF01000414.1+	11576	11643	UUGUAGUCGCACCGCCACCACC	
	NBAF01000521.1+	8369	8436	UUGUAGUCGCACCGCCACCACC	
	NBAF01001057.1+	15581	15648	UUGUAGUCGCACCGCCACCACC	
Novel-miR27	NBAF01001644.1–	306572	306650	AAAUCUUGUACCAAAUUGACCAUG	0.973372858
	NBAF01000262.1–	9795	9872	AAAUCUUGUACCAAAUUGACCAUG	
	NBAF01001559.1–	6457	6535	AAAUCUUGUACCAAAUUGACCAUG	
	NBAF01001299.1+	22721	22799	AAAUCUUGUACCAAAUUGACCAUG	
Novel-miR28	NBAF01001706.1+	99463	99512	UGGUAACGUUGUUCAUUGACAGG	0.689472441
Novel-miR29	NBAF01001572.1+	143059	143121	AGCUUACGACCAUAUCACG	163.7294262
Novel-miR30	NBAF01001556.1+	12491	12565	UGAAAAGUUGGAGCUGCGAGGCC	6.326923578
Novel-miR31	NBAF01001260.1+	29478	29565	AGGAGAUCCAUGGGUUCAAAGUGG	0.486686429
	NBAF01000619.1–	4368	4455	AGGAGAUCCAUGGGUUCAAAGUGG	
	NBAF01001524.1–	94140	94227	AGGAGAUCCAUGGGUUCAAAGUGG	
	NBAF01001699.1+	566815	566902	AGGAGAUCCAUGGGUUCAAAGUGG	
Novel-miR32	NBAF01001434.1+	28387	28459	UCGAUGAUGGCCAAAACGUUGCG	0.486686429

a UMI_TPM, unique molecular identifiers_ transcript per million.

**FIG 3:**
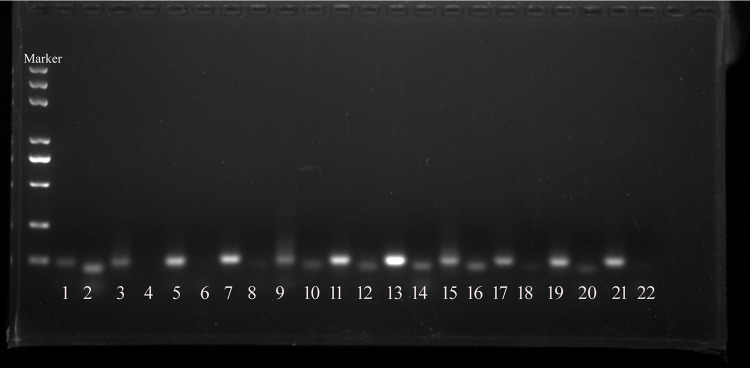
A PCR assay quantified miRNA expression in *D. farinae* exosomes. Lanes 1, 3, 5, 7, 9, 11, 13, 15, 17, and 19 indicate dfa-miR-276, dfa-miR-5735, dfa-miR-252, dfa-miR-1, dfa-let-7b, novel-miR-1, novel-miR-2, novel-miR-3, novel-miR-4 and novel-miR-5, respectively. Lanes 2, 4, 6, 8, 10, 12, 14, 16, 18, and 20 serve as negative controls of the corresponding miRNA, and there is no template in these PCR systems. Lane 21 is an external positive-control cel-miR-39 for all of these PCR systems, and lane 22 is a negative control for lane 21.

Next, PCR assay was used to confirm the presence of these conserved and novel miRNA. The template was transcribed from exosomal RNA by using a poly(A)-tailing method, and a PCR assay was performed to quantify the expression of dfa-miR-276, dfa-miR-5735, dfa-miR-1, dfa-let-7b, novel-miR-1, novel-miR-2, novel-miR-3, novel-miR-4, and novel-miR-5 in *D. farinae* exosomes (Fig. 3).

### Immunoreactivity of *D. farinae* exosomes to serum IgE from allergic patients.

**FIG 4:**
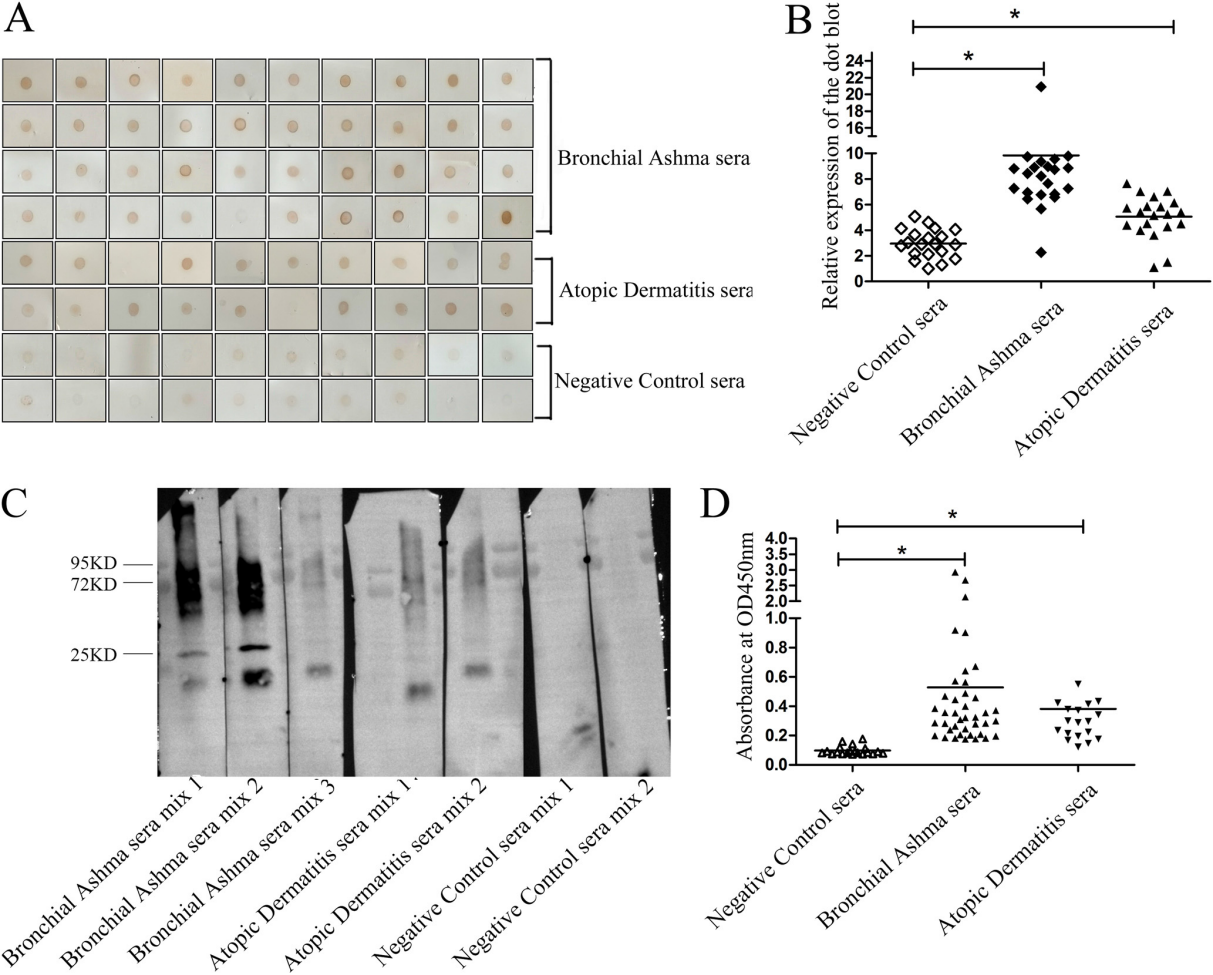
Immunological characterization of *D. farinae* exosomes. Immunoblotting (A and B), Western blotting (C), and ELISA (D) were used to measure the immunoreactivity of *D. farinae* exosomes to serum IgE from *D. farinae*-allergic patients.

To determine the immunoreactivity of *D. farinae* exosomes, immunoblotting was performed using serum samples from 40 children with bronchial asthma and 20 children with atopic dermatitis tested positive for *D. farinae*-specific IgE. The serum IgE from 39 of 40 (97.5%) children with bronchial asthma and 18 of 20 (90.0%) children with atopic dermatitis had reaction with *D. farinae* exosomes, while no obvious reaction was seen among healthy subjects (Fig. 4A and B). Western blotting and enzyme-linked immunosorbent assay (ELISA) further confirmed the specific immunoreactivity of serum IgE antibody against *D. farinae* exosomes (Fig. 4C and D). Compared to the serum that tested negative for *D. farinae*-specific IgE, a higher IgE reactivity against *D. farinae* exosomes was detected in serum that tested positive for *D. farinae*-specific IgE. Our data indicate that *D. farinae* exosomes, as an allergen exporter, had a highly immunogenic ability.

### *D. farinae* exosomes induce allergic airway inflammation in a mouse model.

**FIG 5:**
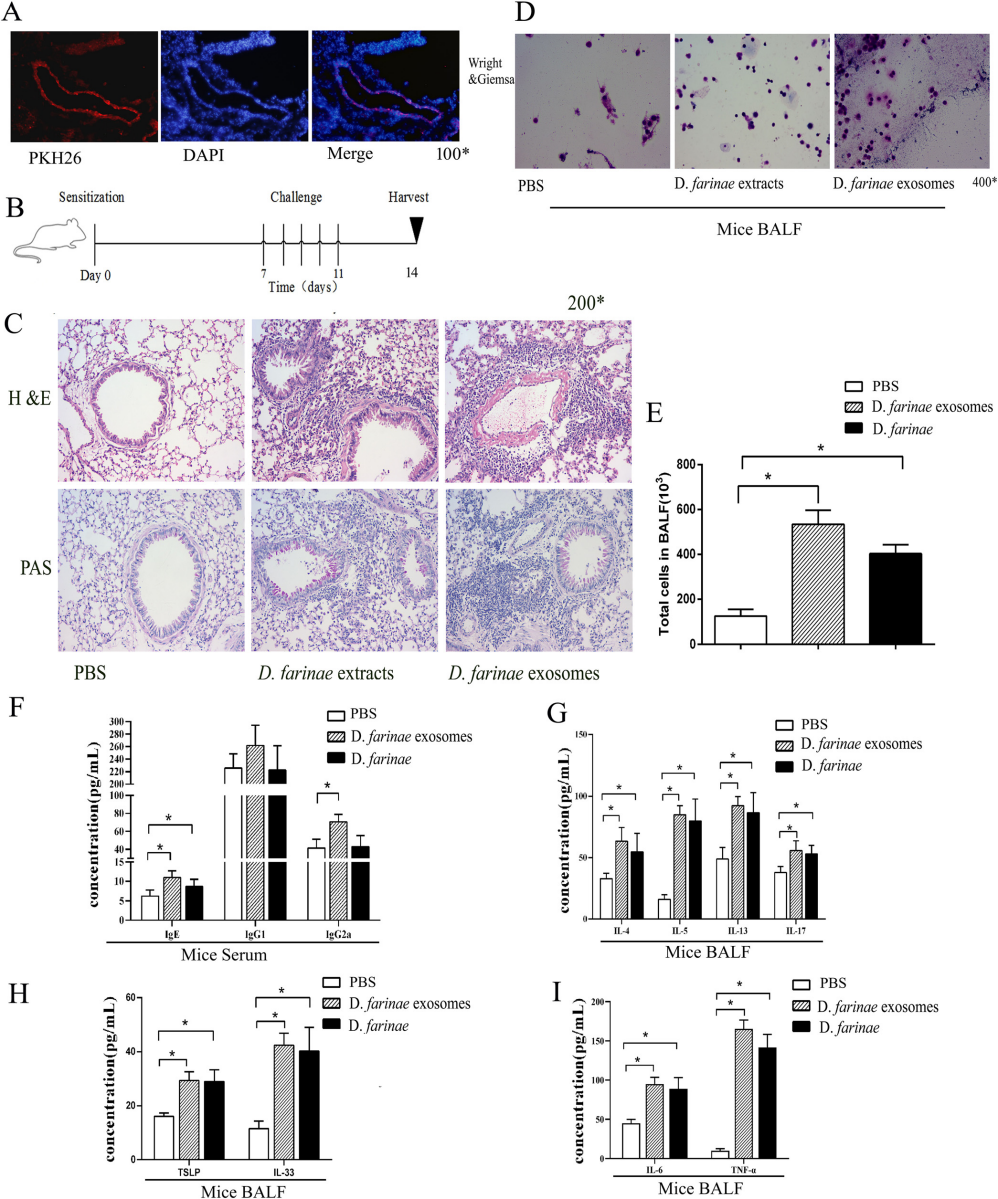
Airway inflammation induced by *D. farinae* exosomes. (A) Uptake of PKH26-labeled *D. farinae* exosomes by mouse airway. (B) Protocol for modeling allergic airway inflammation in mice. (C) Paraffin-embedded lung sections are stained with HE (upper panel) and PAS (lower panel). (D and E) Cell counts in mouse BALF. (F) Serum levels of allergen-specific IgE, IgG1, and IgG2 measured by ELISA. (G, H, and I) Cytokines in BALF measured by ELISA. *, *P < *0.05.

To evaluate the allergenicity of *D. farinae* exosomes, we modeled allergic airway inflammation in a mouse model. First, we tracked the distribution of *D. farinae* exosomes in mice (Fig. 5A), and *D. farinae* exosomes were found in mouse bronchial and lung specimens. Then, mice were immunized with phosphate-buffered saline (PBS), *D. farinae* exosomes, and *D. farinae* extract, respectively (Fig. 5B). Hematoxylin and eosin (HE) and periodic acid-Schiff (PAS) staining of bronchial and lung specimens showed a large number of inflammatory cells surrounding the airways and vessels in *D. farinae* exosomes and *D. farinae* extract groups but not in the PBS group (Fig. 5C). In parallel, the BALF mainly consisted of monocytes, and the total cell numbers increased significantly in the *D. farinae* exosomes and *D. farinae* extract groups relative to the PBS control group (Fig. 5D and E).

ELISA measured higher serum IgE levels in the *D. farinae* exosomes and *D. farinae* extract groups than in the PBS control group (*P < *0.05), and a higher IgG2a level was detected in the *D. farinae* exosomes group than in the PBS control group (*P < *0.05); however, no significant difference was seen among the three groups in terms of serum IgG1 level (Fig. 5F). The representative Th2 cytokines (interleukin-4 [IL-4], IL-5, and IL-13) and Th17 cytokine (IL-17) were measured in BALF. These cytokines were all significantly increased in *D. farinae* exosomes and *D. farinae* extract groups compared to the PBS control group (*P < *0.05) (Fig. 5G). The levels of IL-33 and thymic stromal lymphopoietin (TSLP), representative inflammatory cytokines in bronchoalveolar epithelial cells, were significantly higher in *D. farinae* exosomes and *D. farinae* extract groups than in the PBS control group (*P < *0.05) (Fig. 5H). In addition, tumor necrosis factor alpha (TNF-α) and IL-6, the representative cytokines of alveolar macrophages, were also overexpressed in *D. farinae* exosomes and *D. farinae* extract groups.

### Effects of *D. farinae* exosomes on inflammatory cytokine levels in 16-HBE and NR8383 cells.

**FIG 6:**
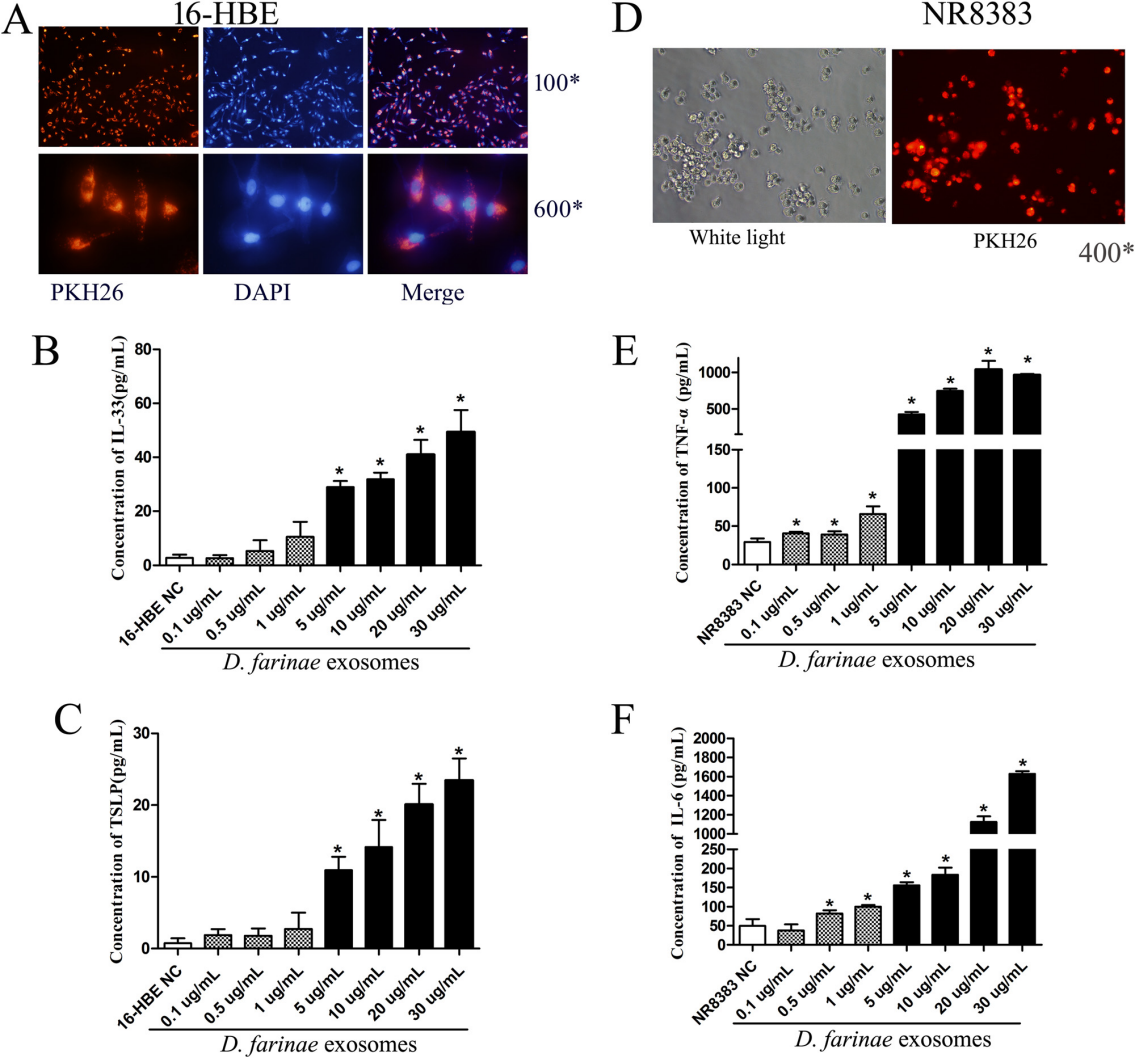
Effects of *D. farinae* exosomes on 16-HBE and NR8383 cells. (A and D) Uptake of PKH26-labeled *D. farinae* exosomes by 16-HBE and NR8383 cells. (B and C) ELISA measures IL-33 and TSLP secreted by 16-HBE cells. (E and F) ELISA measures IL-6 and TNF-α secreted by NR8383 cells. The data show the means ± SD from at least three independent experiments. *, *P < *0.05.

16-HBE and NR8383 cells were used to examine the effect of *D. farinae* exosomes on bronchial epithelial cells and alveolar macrophages. First, 16-HBE and NR8383 cells were found to internalize *D. farinae* exosomes after coincubation (Fig. 6A and D). Then, exposure to *D. farinae* exosomes was found to result in elevated IL-33 and TSLP expression in 16-HBE cells in a dose-dependent manner (Fig. 6B and C). In addition, higher TNF-α and IL-6 levels were also measured in NR8383 cells postexposure to *D. farinae* exosomes in a dose-dependent manner (Fig. 6E and F). Taken together, our data demonstrate that *D. farinae* exosomes may invade bronchial epithelial cells and alveolar macrophages to release inflammation-related cytokines.

### Comparative transcriptomic analysis of 16-HBE and NR8383 cells posttreatment with *D. farinae* exosomes.

**FIG 7:**
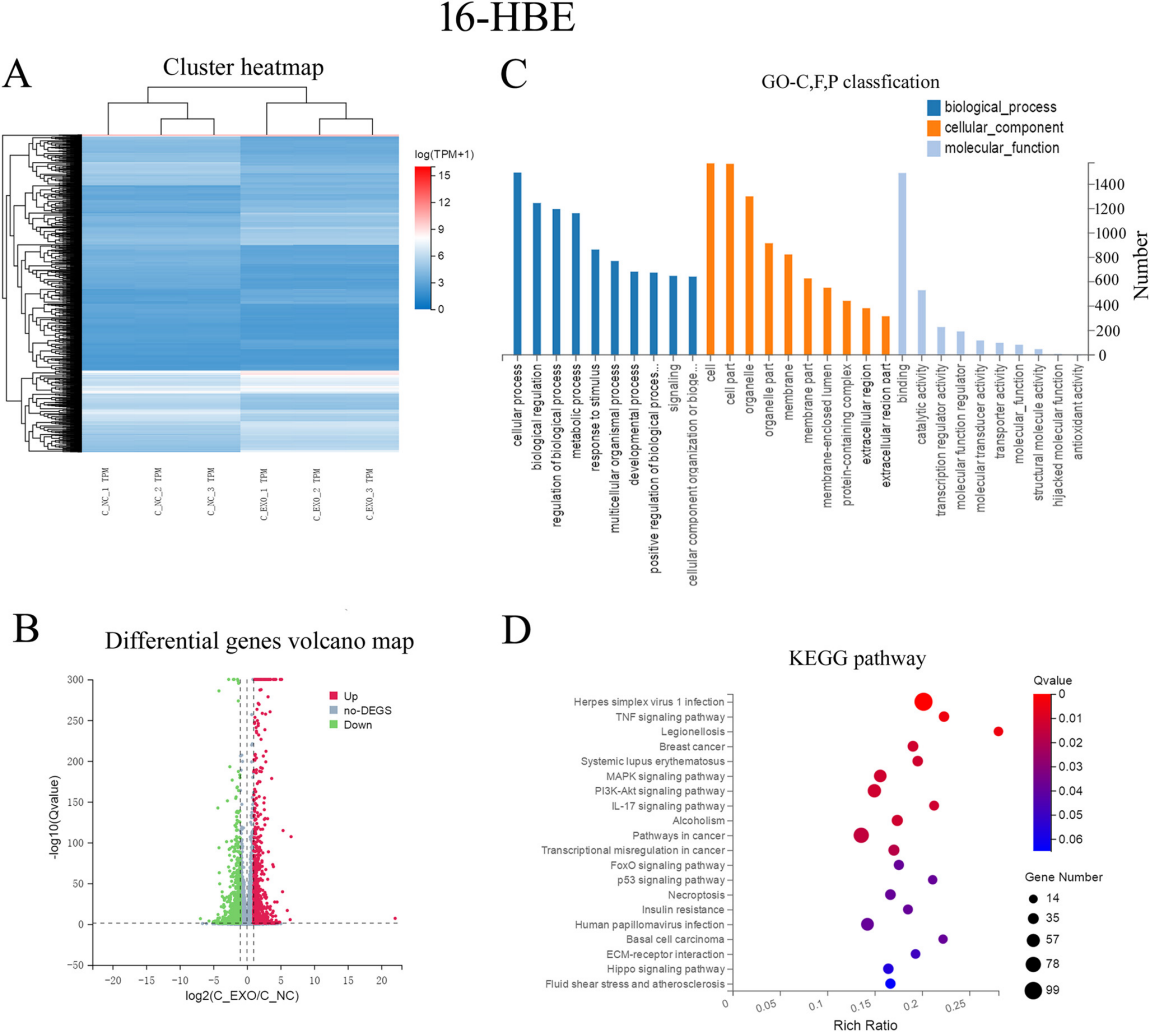
Comparative transcriptomic analysis of 16-HBE cells treated with *D. farinae* exosomes. Differentially expressed genes are defined as a ≥2-fold change and an *F* value of <0.05. (A) Hierarchical clustering of differentially expressed genes between C_NC and C_EXO groups. C_NC group, 16-HBE cells; C_EXO group, 16-HBE cells treated with *D. farinae* exosomes. (B) Volcano plot depicting the profile of differentially expressed genes between C_NC and C_EXO groups. (C and D) GO term and KEGG pathway enrichment analyses of DEGs between 16-HBE cells and 16-HBE cells treated with *D. farinae* exosomes.

**FIG 8:**
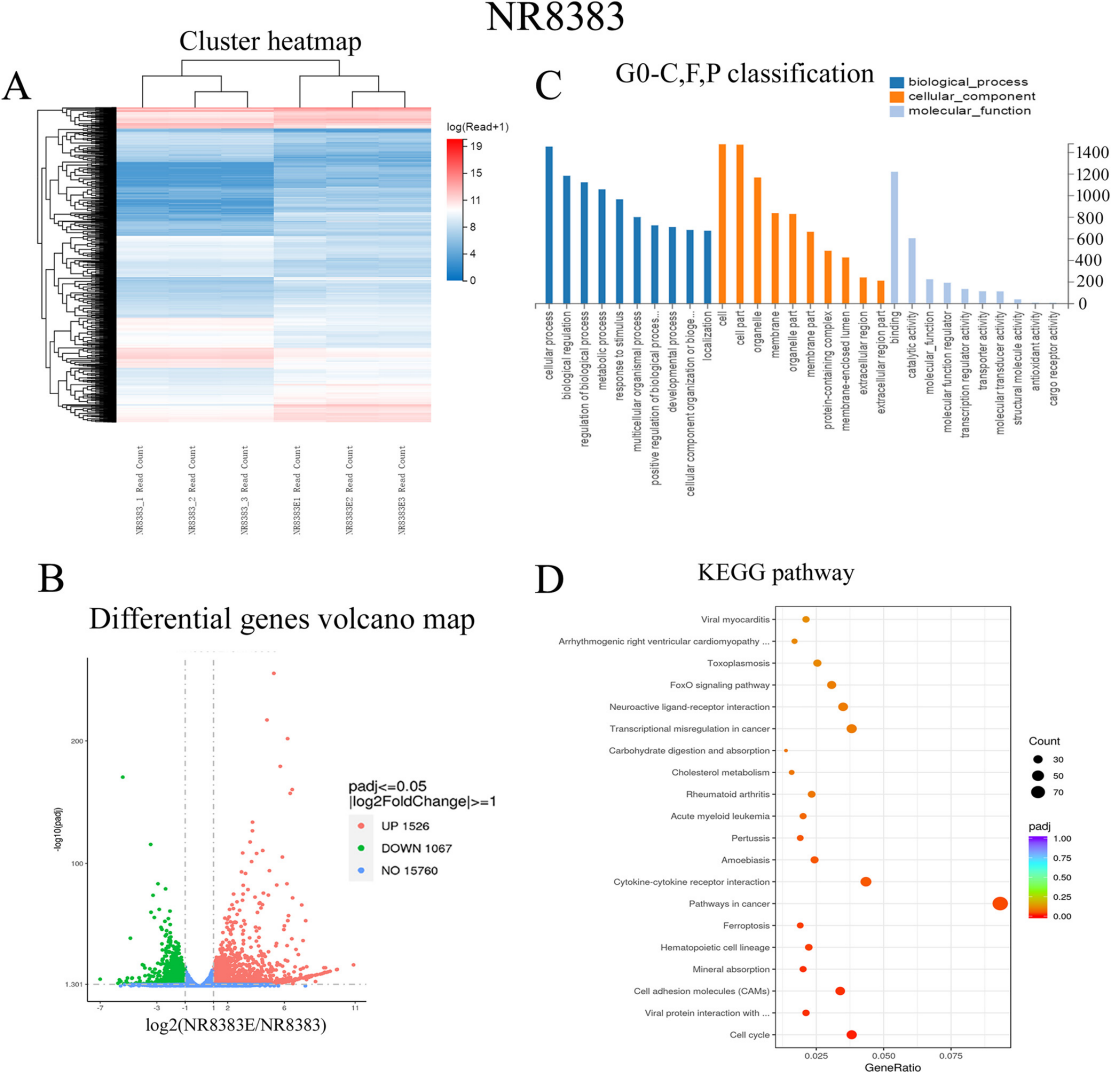
Comparative transcriptomic analysis of NR8383 cells treated with *D. farinae* exosomes. Differentially expressed genes are defined as a ≥2-fold change and an *F* value of <0.05. (A) Hierarchical clustering of DEGs between NR8383 and NR8383E groups. NR8383 group, NR8383 cells; NR8383E group, NR8383 cells treated with *D. farinae* exosomes. (B) Volcano plot depicts the profile of differentially expressed genes between the NR8383 and NR8383E groups. (C and D) GO term and KEGG enrichment analyses of differentially expressed genes between NR8383 cells and NR8383 cells treated with *D. farinae* exosomes.

**FIG 9:**
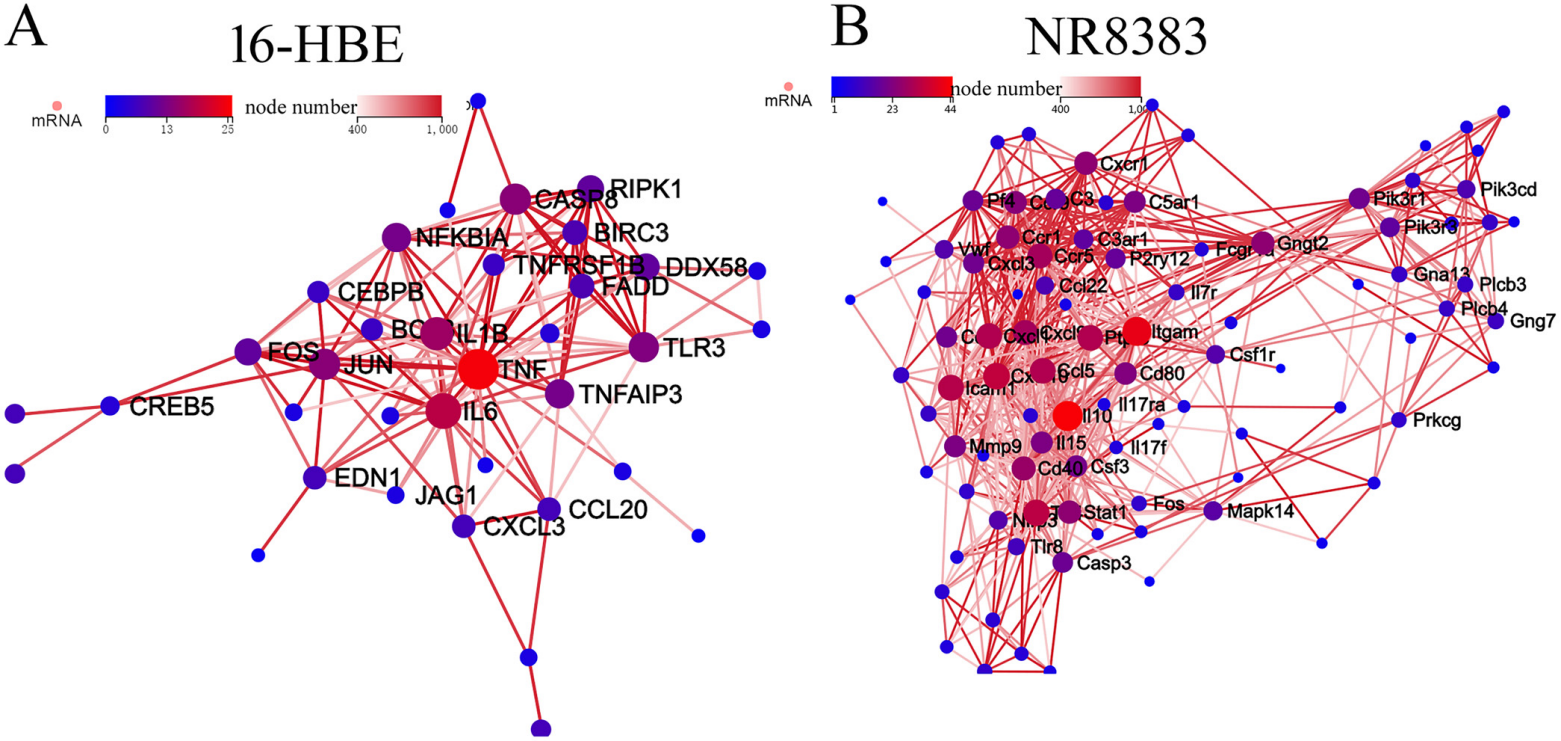
PPI network analysis of the most highly expressed genes in 16-HBE (A) and NR8383 (B) cells treated with *D. farinae* exosomes.

The aforementioned data demonstrated that 16-HBE and NR8383 cells might endocytose *D. farinae* exosomes, and representative allergic inflammatory cytokines were increased in 16-HBE and NR8383 cells after treatment with *D. farinae* exosomes. Therefore, we hypothesized that *D. farinae* exosome-induced allergic asthma might be associated with both 16-HBE and NR8383 cells. To test our hypothesis, a comparative transcriptomic analysis was performed to detect transcriptomic changes of 16-HBE and NR8383 cells after treatment with *D. farinae* exosomes (Fig. 7A and 8A). A total of 1,853 differentially expressed genes were identified in 16-HBE cells after treatment with *D. farinae* exosomes, including 994 downregulated genes and 859 upregulated genes (Fig. 7B), and 2,593 differentially expressed genes screened in NR8383 cells, including 1,067 downregulated genes and 1,526 upregulated genes (Fig. 8B). Gene Ontology (GO) term enrichment analysis showed that the biological processes and cellular components of differentially expressed genes were evenly distributed in 16-HBE and NR8383 cells treated with *D. farinae* exosomes, and the molecular functions of differentially expressed genes were significantly enriched in the binding and catalytic activity (Fig. 7C and 8C). KEGG (Kyoto Encyclopedia of Genes and Genomes) pathway enrichment analysis revealed that the differentially expressed genes of 16-HBE cells treated with *D. farinae* exosomes were significantly enriched in herpes simplex virus 1 (HSV-1) infection, TNF signaling, legionellosis and systemic lupus erythematosus pathways (Fig. 7D). The HSV-1 infection pathway, the most significantly enriched KEGG pathway, was selected for protein-protein interaction (PPI) network analysis, and seven top hub genes were identified, including IL-1β, TNF, IL-6, Toll-like receptor 3 (TLR3), NF-κB, CXCLs, and CCLs (Fig. 9A). The differentially expressed genes of NR8383 cells treated with *D. farinae* exosomes were significantly enriched in cell cycle, viral protein interaction, amoebiasis, rheumatoid arthritis and toxoplasmosis pathways (Fig. 8D), and PPI network analysis of the 100 most highly expressed genes identified six top hub genes, including CXCLs, CCRs, IL-10, IL-5, IL-17, and NLRP3 (Fig. 9B).

## DISCUSSION

The contribution of *D. farinae* allergic airway inflammation has been extensively investigated (30); however, the pathogenic role of exosomes isolated from *D. farinae* in allergic airway inflammation remains unknown until now. To the best of our knowledge, this is the first study to show that exosomes from *D. farinae* trigger allergen-specific immune responses, and we also identified the cargo of *D. farinae* exosomes and preliminarily unraveled the possible sensitization mechanisms of *D. farinae* exosomes in airway allergic inflammation.

The effect of exosomes on mammalian cells has been extensively examined; however, few studies have focused on exosomes derived from invertebrates (12). Extracellular vesicles isolated from crude HDM extracts, which originated from Gram-negative bacteria *Bacteroidetes* and *Proteobacteria*, were found to trigger innate immune responses (31), and intranasal administration of HDM-derived extracellular vesicles induced production of cytokines associated with development of mixed Th1/Th2/Th17-mediated airway inflammation in naive mice, suggesting that HDM-derived extracellular vesicles contributed to the development of airway inflammation (31). In the present study, *D. farinae* exosomes were assumed to be derived from mite bodies, secretions, excretions, and even from its habitat microbiomes, resulting in a high diversity of acquired exosomes. This is the first study to characterize *D. farinae* exosomes, and further studies to unravel the exact functions of *D. farinae* exosomes seem justified.

Exosomes are vesicles generated by different cells that carry nucleic acids, proteins, lipids, and metabolites (32). In this study, 29 proteins identified using shotgun LC-MS/MS are allergens recognized by WHO/IUIS Allergen Nomenclature Subcommittee. There are two possible reasons to explain why *D. farinae* exosomes are abundant in allergens: (i) the exosomes we extracted are derived from different tissues, and (ii) the allergen structures are explored more detailed in UniProt database. These finding implies that *D. farinae* exosomes may be present in a form of allergic ingredient in addition to mite bodies, secretions and excretions. In addition, GAPDH, aquaporin, HSP70, and other characteristic proteins of exosomes were also identified, which further confirm that the microvesicles we acquired are rich in exosomes. Notably, we identified the protein of *Cardinium* in *D. farinae* exosomes. *Cardinium* spp. were reported to be involved in alterations of the reproductive system in arthropod hosts, such as cytoplasmic incompatibility, parthenogenesis, and feminization (33). We hypothesized that *Cardinium* may regulate the biological functions of *D. farinae* or be involved in sensitization through wrapping in *D. farinae* exosomes. Correlation analyses between host and symbiont gene expression profiles revealed that the expression of *Cardinium* genes explained 95% of the variation in the expression of mite genes assigned to phagocytosis, apoptosis, MAPK signaling cascade, endocytosis, TNF, TGF-β, lysozyme, and Toll/Imd pathways, and the expression of mite genes explained 76% of the variability in *Cardinium* gene expression, while the expression of the *Cardinium* genes encoding the signaling molecules BamD, LepA, SymE, and VirD4 was associated with the expression levels of mite genes involved in endocytosis, phagocytosis, and apoptosis (34). Our data suggest that exosomes may serve as a communication between *D. farinae* and *Cardinium*.

The significantly upregulated miRNAs of *D. farinae* exosomes identified by RNA-Seq analysis were also highly expressed in other arthropods (29, 35). As the most abundant miRNA, miR-276 was reported to promote developmental synchrony in *Locusta migratoria* (36) and to shape the mosquito reproductive cycle and Plasmodium falciparum development through modulating metabolic balance (37). This is the first study to present high miR-276 expression in *D. farinae*, and further studies to investigate the pathogenic role of miR-276 in allergic diseases are warranted. In addition, the targets of miR-5735-5p have been identified in *D. pteronyssinus*, and the targeted genes were found to be enriched in allergy-related pathways (28). miR-1-3p has been also identified as an allergy-regulating gene that plays an important role in the development of asthma (26). In the present study, 9 of the 32 novel miRNAs were quantified to have expression levels of >1,000 transcripts per million, implying that novel miRNAs have an important role in the biologic function of *D. farinae* exosomes.

Here, the immunogenicity of *D. farinae* exosomes to sera from children with allergic bronchial asthma and atopic dermatitis were found to present a high IgE-binding capacity. In addition, we found that the expression of Th2 representative inflammatory cytokines, such as IL-4, IL-5, and IL-13, was increased in a mouse model inhaled with *D. farinae* exosomes. In addition, high expression of IL-17, a representative cytokine of Th17, was also detected. Th1/Th2 imbalance is a major contributor to allergic diseases, and Th17 immune response is also involved in allergic diseases (38, 39). Our data imply that both Th2 and Th17 immune responses participate in allergic airway disease triggered by *D. farinae* exosomes.

As the first line of the respiratory system, airway epithelial cells and alveolar macrophages have an important role in environmental allergen-induced airway inflammation (40, 41). Our data showed that the airway epithelial cells and alveolar macrophage endocytosed *D. farinae* exosomes and showed high expression of representative cytokines. These data suggest that airway epithelial cells and alveolar macrophages play an important role in *D. farinae* exosome-mediated airway inflammation. Then, our comparative transcriptomic analysis of 16-HBE and NR8383 cells identified that immune pathways and immune cytokines/chemokines were involved in the sensitization of *D. farinae* exosomes. For example, TLR, the main pattern recognition receptor of allergen protein, was upregulated, and its downstream regulating proinflammatory cytokines/chemokines were increased in 16-HBE cells, including IL-6, TNF-α, CCLs, and CXCLs (42). These cytokines/chemokines may induce asthma by attracting and/or activating cells from the innate and adaptive immune system (40). In NR8383 cells, CCLs and CXCLs were found to be upregulated, which may also contribute to asthma and other inflammation-related disease (41).

Previous studies have shown that allergens may be inhaled via adhering fecal particles (9). In HDMs, the primary allergen-containing particles are fecal waste pellets, and these particles—20 to 25 μm in size—are approximately the same size as pollen grains, while particles containing mite allergens are generally much heavier and therefore quicker to settle to the ground (43). *D. farinae* exosomes, which are rich in allergens and 150 nm in diameter, may represent a better HDM allergen transport carrier, since they may float in the air for a long period of time and can be more easily absorbed by effector cells in lungs. It is therefore hypothesized that if *D. farinae* exosomes are inhaled by bronchi, an immediate immune response may occur, leading to wheezing, dyspnea, cough, and chest tightness.

In summary, the results of the present study demonstrate that *D. farinae* exosomes are immunogenic and may induce allergic airway inflammation via bronchial epithelial cells and alveolar macrophages. Further studies to unravel the mechanisms underlying allergic airway inflammation induced by *D. farinae* exosomes are warranted.

## MATERIALS AND METHODS

### *D. farinae* specimens.

*D. farinae* was cultured at 25°C in an air-filtered room with 70% relative humidity. A culture medium consisting of yeast, starch, and rice flour was baked at 80°C for 30 min and then used for the large-scale mite cultivation. Two experiments were used to identify the purity of *D. farinae*. (i) First, *D. farinae* was collected with an acupuncture needle from the culture medium and transferred onto a slide with a drop of Hoyer’s medium (Shifeng Biological Corporation, Shanghai, China). The mite-loaded slides were then observed under a light microscope (Olympus Corporation, Tokyo, Japan). (ii) Second, a nested PCR assay was performed to distinguish *D. farinae* from *D. pteronyssinus*, as previously described (44).

### Cell lines and culture.

Human bronchial epithelial 16-HBE cell line and rat alveolar macrophage NR8383 cell line were purchased from Procell Biological Company (Shanghai, China) and cultured in Dulbecco modified Eagle medium (Thermo Fisher Scientific, Grand Island, NY) supplemented with 10% fetal bovine serum (Sigma-Aldrich, St. Louis, MO) and 1% penicillin-streptomycin (Sigma-Aldrich) in a humidified atmosphere containing 5% CO_2_ at 37°C.

### Animals.

The C57BL/6 mouse models of asthma were purchased from Center for Laboratory Medicine, Yangzhou University (Yangzhou, China). All animals were housed in a pathogen-free environment with a temperature of 22 ± 1°C, a relative humidity of 50% ± 1%, and a 12/12-h light/dark cycle.

### Isolation and identification of exosomes from *D. farinae*.

Purified *D. farinae* (20 g) was washed three times using ddH_2_O, resuspended in 100 mL of cold, sterile 1× PBS (Gibco, Grand Island, NY), and then stirred overnight at 4°C. The mixture was filtered through a 40-μm-pore-size cell strainer (Biologix, Camarillo, CA) and centrifuged at 3,500 × *g* for 20 min, and the supernatant was harvested. Next, the supernatant was centrifuged at 10,000 × *g* for 1 h at 4°C and filtered through a 0.22-μm-pore-size cell strainer (Millipore, Burlington, MA) to remove debris, followed by centrifugation at 120,000 × *g* for 2 h at 4°C to yield light-yellow gel-like precipitates. Subsequently, 20 mL of sterile PBS was further used to wash the light-yellow gel-like precipitates, and a second ultracentrifugation was performed under the same condition to acquire higher-purity gel-like precipitates. The acquired pellets were then resuspended in 1 mL of sterile PBS, and the protein content was measured using a BCA protein assay kit (Beyotime, Shanghai, China) after lysis with a radioimmunoprecipitation assay (Beyotime) in a volume of 4:1.

The morphology of *D. farinae* extracellular vesicles was characterized using a JEM-1400 transmission electron microscope (JEOL, Tokyo, Japan). The particle size of *D. farinae* exosomes was measured with a ZetaView PMX110 multiple parameter particle tracking analyzer (Particle Metrix, Meerbusch, Germany). Three video cycles were recorded, and the results were analyzed using ZetaView 8.04.02 SP2 software (Particle Metrix). In addition, the signature proteins HSP70 and CD63 of *D. farinae* exosomes were determined between exosomes and dust mite body using a Western blotting assay, and the amount of protein loaded in each sample was 10 μg.

### Identification of proteins in *D. farinae* exosomes using shotgun LC-MS/MS.

The *D. farinae* exosomal peptide samples were subjected to LC-MS/MS analysis using a Q EXactive mass spectrometer coupled with an Easy nLC 1200 system (Gengchem, Shanghai, China). The acquired raw data were imported into the software Protein Discoverer 2.2 for protein identification and quantification, followed by a database search using Mascot 2.6 engines. Protein identification was performed in UniProt and NCBI databases with search parameters permitting with a maximum of two missed cleavages. A precursor mass tolerance of 10 ppm was specified, as well as a 0.05-Da tolerance for MS fragments. Protein was considered positively identified if a specific peptide score reached a threshold false discovery rate (FDR) of <0.01.

### Identification of miRNAs in *D. farinae* exosomes using sRNA-Seq.

The miRNAs in *D. farinae* exosomes were sequenced with the BGISEQ-500 sequencer (BGI, Shenzhen, China). The detailed sequencing procedures are described in Fig. S1 in the supplemental material. After the removal of low-quality reads, a total of 46,095,191 clean reads were generated and submitted to the NCBI (BioProject PRJNA945179). The length distribution of small RNA is presented in Fig. S2. After filtration, clean reads were mapped to the miRBase database (http://www.mirbase.org/). The identified miRNAs were categorized into two groups (conserved and novel miRNAs) based on whether they were exactly matched to currently known miRNAs in the miRBase database. If *D. farinae* miRNA was identical to at least one of the miRNAs in the miRBase database, the miRNA was defined as a conserved miRNA; otherwise, the identified miRNA was considered a novel miRNA and characterized using the miRDeep2 package according to the mature sequences and predicted hairpin sequences of this species.

### Assessment of the immunogenicity of *D. farinae* exosomes.

Serum samples were collected from 40 children with allergic bronchial asthma, and 20 children with atopic dermatitis tested positive for *D. farinae*-specific IgE with CAP System (45) (Pharmacia & Upjohn Diagnostics AB, Bridgewater, NJ), while serum samples from 20 healthy children negative for *D. farinae*-specific IgE served as controls. Participant demographic and clinical characteristics are demonstrated in Table S1.

For Western blotting assay, the protein (10 μg/lane) of *D. farinae* exosomes was separated and transferred to polyvinylidene difluoride membranes. Subsequently, the blots were blocked and incubated with the mixture of allergic children’s sera and controls (1:10 [vol/vol]; the concentration was also used for dot blotting and ELISAs). After a wash in PBS, the membrane was incubated with horseradish peroxidase-conjugated mouse anti-human IgE secondary antibody (1:2,000 [Invitrogen, Waltham, MA]; the same concentration was used for dot blotting and ELISAs). The blots were visualized using enhanced chemiluninescence.

For the dot blotting assay, 0.5 μg of protein of *D. farinae* exosomes was added to the nitrocellulose membrane (Millipore) to detect the IgE antibody. The dots were visualized with a diaminobenzidine horseradish peroxidase color development kit (Beyotime).

For ELISAs, each well was coated with 0.2 μg of protein extracted from *D. farinae* exosomes. Each well was blocked and incubated with sera. Then, each well was incubated with anti-human IgE secondary antibody and detected with TMB (3,3′,5,5′-tetramethylbenzidine). The reaction was stopped, and the absorbance was read with a microplate reader (Bio-Rad, Irvine, CA).

### *D. farinae* exosome tracking.

*D. farinae* exosomes were labeled with PKH26 cell linker kit for general cell membrane labeling (BestBio, Shanghai, China). Briefly, the PKH26 dye was mixed with *D. farinae* exosomes and incubated. The mixture was then added with 10 mL of PBS, transferred to 100-kDa ultrafiltration tubes (Millipore), and centrifuged at 1,000 × *g* for 30 min. The purified, labeled exosomes were added into cells or intranasally administered to C57BL/6 mice and then observed using IX71 fluorescence microscopy (Olympus).

### Modeling allergic airway inflammation in mice.

A total of 24 C57BL/6 female mice, 6 to 8 weeks old, were randomly divided into three groups of 8 mice. The *D. farinae* extract and exosome groups were both administered intranasally with 40 μL of *D. farinae* extract (Greer Laboratories, Inc., Lenoir, NC) at a concentration of 2.5 μg/μL on day 0. Mice in the *D. farinae* extract group were administered intranasally with 10 μg of *D. farinae* extract since day 7, once daily for 5 days, and mice in the *D. farinae* exosome group were administered intranasally with 10 μg of *D. farinae* exosomes once daily for 5 days, while animals given PBS served as controls. Then, BALF was sampled, and cell numbers were counted. Cytokine levels in BALF and serum samples were measured using ELISA with commercial reagent kits (Mlbio, Shanghai, China). The lung specimens were sampled, fixed with 4% paraformaldehyde, dehydrated, embedded in paraffin, cut into sections with 4 μm in thickness, and stained with HE and PAS solutions for histopathological analysis.

### Cell treatments and RNA-Seq.

16-HBE and NR8383 cells were harvested 24 h after single exposure to 30 μg/mL *D. farinae* exosomes, and three independent samples were examined after each exposure to *D. farinae* exosomes or PBS. Total RNA was extracted and sequenced on a BGISEQ-500 platform (BGI) and an Illumina Novaseq X Plus system platform (Illumina, Inc., San Diego, CA). Three samples were analyzed for each group as a single cluster to determine the level of similarity within the group. The numbers of clusters were independently assessed using hierarchical clustering to construct a heat map. After RNA-Seq, the relative gene expression level was quantified using RNA-Seq expression estimation by expectation-maximization (RSEM) software, version 1.3.1 (46), and a heatmap was plotted using the package pheatmap version 1.0.8 (47) according to the gene expression difference in various samples. Differential expression analysis was performed using the software DESeq2 version 1.4.5 (48). Genes that changed at least 2-fold before and after treatment were compared (*D. farinae* exosomes versus PBS treatment), and those with an FDR < 0.05 were used to construct a volcano map. To assess the change of phenotype, more than twice the differentially expressed genes (DEGs) with an FDR of <0.05 were subjected to Gene Ontology (GO) term (http://www.geneontology.org/) and KEGG pathway enrichment analyses (https://www.kegg.jp/). GO and KEGG enrichment analyses of annotated DEGs were performed using the phyper function of the R package (https://en.wikipedia.org/wiki/Hypergeometric_distribution) based on a hypergeometric test. The significant levels of terms and pathways were corrected by Q value with a rigorous threshold (Q ≤ 0.05).

### Statistical analysis.

All measured data are presented as means ± the standard deviations (SD), and the differences in means were compared between groups by using a Student *t* test. All statistical analyses were performed using Prism software (version 5; GraphPad Software, San Diego, CA), and a *P* value of <0.05 was indicative of statistical significance.

### Ethical approval.

This study was approved by the Institutional Ethical Review Committee of Wuxi 2 People’s Hospital (approval 2022-Y-76). Written informed consent was obtained from all participants following a detailed description of the purpose of the present study. Animal experiments were performed strictly following the 3R principle and international and national laws, regulations and guidelines for the care and management of laboratory animals.

### Data availability.

The sRNA-seq data presented here were deposited in the NCBI database under BioProject accession number PRJNA945179.
