# Supplementary material for: Exosomes Derived from Dermatophagoides farinae Induce Allergic Airway Inflammation
Source: Microbiol Spectr. 2023 Jun 14;11(4):e05054-22. doi: 10.1128/spectrum.05054-22 (PMC10434197; doi:10.1128/spectrum.05054-22)
Supplement: Supplemental file 1 — Supplemental materials and methods, Fig. S1 to S3, and Table S1. Download spectrum.05054-22-s0001.pdf, PDF file, 0.3 MB [file spectrum.05054-22-s0001.pdf]

Supplementary Materials

Material and methods

Experiment pipeline and quality control

Each step in the experiment pipeline (from sample testing, library construction to sequencing) influences the data quality and quantity, this then directly affects bioinformatics analysis results. In order to get reliable sequencing data, we carry out strict quality control at each step of the experiment. The experiment pipeline was described in Figure S1.

The raw sequencing data are called raw tags. The raw tags were processed using the following steps: removing low quality tags, removing tags with 5 primer contaminants, removing tags without 3 primer, removing tags without insertion, removing tags with poly A and removing tags shorter than 18 nt. 46,095,191 clean tags were carried out and the length distribution of sRNA was also displayed in Figure S2.

GO and KEGG enrichment analysis of differentially expressed genes

Gene Ontology (GO) enrichment analysis of differentially expressed genes was implemented by the clusterProfiler R package, in which gene length bias was corrected. GO terms with corrected P value less than 0.05 were considered significantly enriched by differential expressed genes.

KEGG is a database resource for understanding high-level functions and utilities of the biological system, such as the cell, the organism and the ecosystem, from molecular-level information, especially large-scale molecular datasets generated by genome sequencing and other high-through put experimental technologies (http://www.genome.jp/kegg/). We used clusterProfiler R package to test the statistical enrichment of differential expression genes in KEGG pathways.

Fig. S1 Experiment pipeline: from sample testing, library construction to sequence

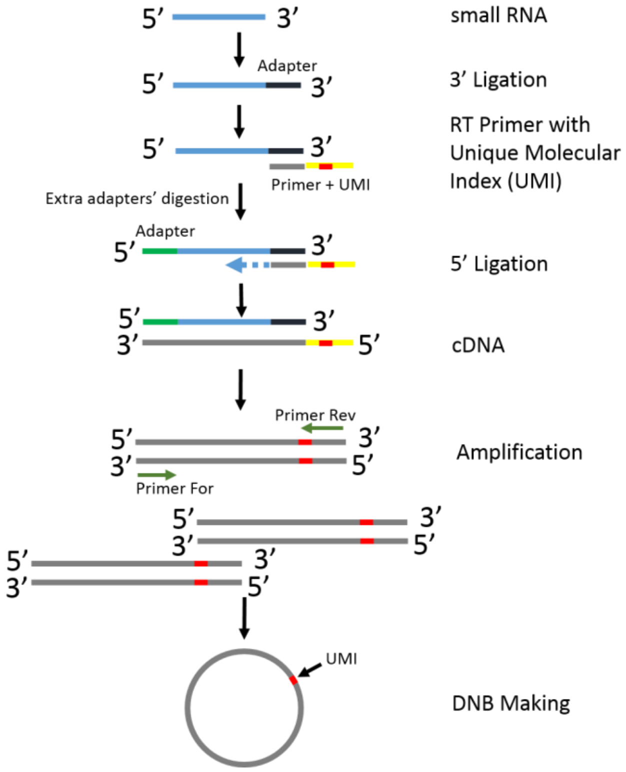

Fig. S2 Length distribution of sRNA: the X axis showed the length of sRNA and the Y axis showed the percentage of the number of sRNA with specific length.

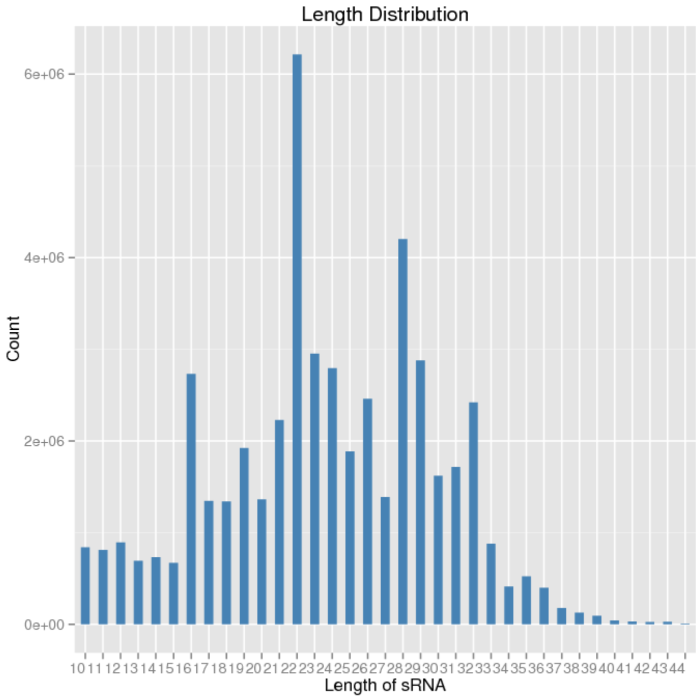

Fig. S3 Detection *Cardinium* endosymbiont in *D. farinae*. A, 16S rRNA of *Cardinium* was detected by PCR. Line 1-4: DNA templates from *D. farinae*; Line 5-6: DNA templates from *D. pteronyssinus*. B, Blast result of PCR product sequence from Line 1-4.

A

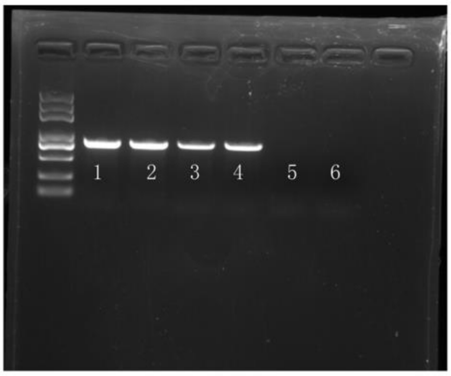

B

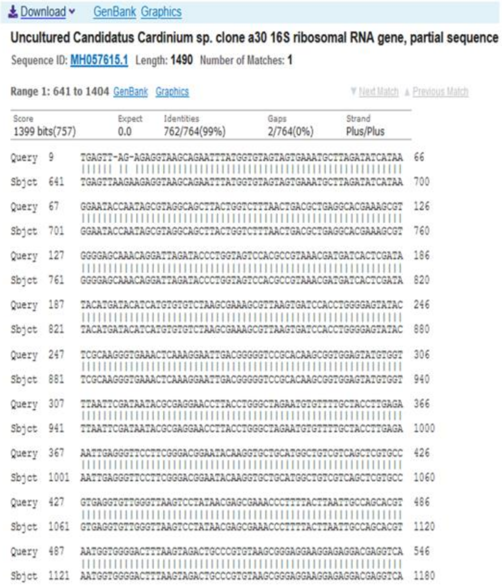

Table S1 The clinical features of 80 participants providing serum samples

Patients	Age(years)	Male/Female	Clinical history	Allergy to D. farinae	Allergy to D. pteronyssinus	[#]IgE ((KUA/L)
1	3	Male	BA[#]	+	+	>100/1.64
2	4	Female	BA	+	+	>100/55.6
3	5	Female	BA	+	+	>100/5.7
4	5	Male	BA/AR[#]	+	−	90.9/0.13
5	4	Male	BA	+	+	>100/33.8
6	8	Female	BA/AR	+	+	>100/93.2
7	5	Male	BA	+	+	>100/1.75
8	2	Male	BA	+	+	81.7/52.7
9	10	Male	BA	+	−	>100/0.17
10	4	Female	BA	+	+	>100/35.4
11	4	Male	BA	+	+	>100/1.29
12	5	Male	BA/AR	+	−	>100/0.13
13	4	Male	BA	+	+	>100/3.33
14	3	Male	BA	+	+	>100/67.1
15	6	Male	BA	+	−	>100/0.13
16	4	Male	BA	+	+	>100/>100
17	3	Female	BA	+	−	>100/0.13
18	6	Female	BA	+	+	>100/0.77
19	8	Female	BA	+	+	>100/>100
20	8	Male	BA/AR	+	+	71.5/1.32
21	4	Female	BA	+	−	99.6/0.14
22	3	Male	BA	+	+	100/100
23	3	Male	BA	+	+	80.2/78.6
24	4	Male	BA	+	+	100/100
25	11	Male	RA	+	+	100/65.4
26	6	Male	BA	+	+	100/54.9
27	8	Male	BA	+	+	100/70.6
28	2	Male	RA	+	+	100/100
29	10	Male	RA	+	+	100/100
30	3	Male	BA	+	+	100/76.3
31	4	Female	BA	+	+	100/100
32	4	Female	BA	+	+	100/100
33	4	Male	BA	+	+	100/100
34	5	Male	BA	+	+	100/100
35	8	Female	BA	+	+	100/89.3
36	5	Male	BA	+	+	100/34.3
37	3	Female	BA	+	+	100/49.3
38	4	Male	BA	+	+	97/100
39	4	Male	BA	+	+	100/100
40	5	Male	BA	+	+	>100/>100

41	3	Male	AD[#]	+	+	95.3/>100
42	8	Male	AD	+	+	>100/>100
43	9	Male	AD	+	-	>100/9.12
44	8	Male	AD	+	-	>100/0.16
45	13	Male	AD	+	+	>100/>100
46	7	Male	AD	+	-	64.4/34.60
47	4	Male	AD	+	+	>100/>100
48	10	Male	AD	+	+	>100/>100
49	4	Female	AD	+	+	>100/56.2
50	8	Male	AD	+	+	>100/>100
51	11	Male	AD	+	+	94.7/62.5
52	8	Female	AD	+	+	>100/76.4
53	3	Male	AD	+	+	83.9/65
54	13	Female	AD	+	+	100/55.3
55	10	Male	AD	+	+	100/65.7
56	9	Male	AD	+	+	100/100
57	4	Male	AD	+	+	100/84.3
58	5	Female	AD	+	+	83.3/92.4
59	4	Male	AD	+	+	100/100
60	6	Male	AD	+	+	90.7/1.02
61	6	Male	NC[#]	-	-	0.04/0.06
62	6	Male	NC	-	-	0.01/0.13
63	2	Female	NC	-	-	0.02/0.13
64	4	Female	NC	-	-	0.03/0.11
65	3	Female	NC	-	-	0.11/0.11
66	4	Male	NC	-	-	0.14/0.11
67	4	Female	NC	-	-	0.10/0.14
68	1	Female	NC	-	-	0.02/0.05
69	3	Male	NC	-	-	0.03/0.15
70	4	Male	NC	-	-	0.02/0.15
71	4	Female	NC	-	-	0.11/0.05
72	4	Male	NC	-	-	0.03/0.14
73	3	Female	NC	-	-	0.13/0.11
74	5	Male	NC	-	-	0.12/0.12
75	10	Male	NC	-	-	0.13/0.11
76	4	Female	NC	-	-	0.03/0.06
77	3	Female	NC	-	-	0.02/0.04
78	4	Female	NC	-	-	0.02/0.04
79	5	Male	NC	-	-	0.20/0.24
80	5	Male	NC	-	-	0.02/0.04

BA: bronchial asthma; AR: allergic rhinitis; AD: atopic dermatitis; NC: Negative control; [#]IgE ((KUA/L): IgE specific to *D. farinae*/ IgE specific to D. *pteronyssinus*
